# Hybrid phenolic-inducible promoters towards construction of self-inducible systems for microbial lignin valorization

**DOI:** 10.1186/s13068-018-1179-8

**Published:** 2018-06-28

**Authors:** Arul M. Varman, Rhiannon Follenfant, Fang Liu, Ryan W. Davis, Yone K. Lin, Seema Singh

**Affiliations:** 1 Biomass Science and Conversion Technology Department, Sandia National Laboratories, Livermore, CA USA 94550; 2 Joint Bioenergy Institute, Emeryville, CA USA 94608; 3 Department of Bioproducts and Biosystems Engineering, University of Minnesota, St. Paul, MN 55108 USA; 4 Chemical Engineering, School for Engineering of Matter, Transport, and Energy, Arizona State University, Tempe, AZ 85287 USA

**Keywords:** Lignin valorization, Promoter engineering, Biochemicals, Hybrid promoters, Phenolic inducers

## Abstract

**Background:**

Engineering strategies to create promoters that are both higher strength and tunable in the presence of inexpensive compounds are of high importance to develop metabolic engineering technologies that can be commercialized. Lignocellulosic biomass stands out as the most abundant renewable feedstock for the production of biofuels and chemicals. However, lignin a major polymeric component of the biomass is made up of aromatic units and remains as an untapped resource. Novel synthetic biology tools for the expression of heterologous proteins are critical for the effective engineering of a microbe to valorize lignin. This study demonstrates the first successful attempt in the creation of engineered promoters that can be induced by aromatics present in lignocellulosic hydrolysates to increase heterologous protein production.

**Results:**

A hybrid promoter engineering approach was utilized for the construction of phenolic-inducible promoters of higher strength. The hybrid promoters were constructed by replacing the spacer region of an endogenous promoter, *P*_emrR_ present in *E. coli* that was naturally inducible by phenolics. In the presence of vanillin, the engineered promoters *P*_vtac_, *P*_vtrc_, and *P*_vtic_ increased protein expression by 4.6-, 3.0-, and 1.5-fold, respectively, in comparison with a native promoter, *P*_emrR_. In the presence of vanillic acid, *P*_vtac_, *P*_vtrc_, and *P*_vtic_ improved protein expression by 9.5-, 6.8-, and 2.1-fold, respectively, in comparison with *P*_emrR_. Among the cells induced with vanillin, the emergence of a sub-population constituting the healthy and dividing cells using flow cytometry was observed. The analysis also revealed this smaller sub-population to be the primary contributor for the increased expression that was observed with the engineered promoters.

**Conclusions:**

This study demonstrates the first successful attempt in the creation of engineered promoters that can be induced by aromatics to increase heterologous protein production. Employing promoters inducible by phenolics will provide the following advantages: (1) develop substrate inducible systems; (2) lower operating costs by replacing expensive IPTG currently used for induction; (3) develop dynamic regulatory systems; and (4) provide flexibility in operating conditions. The flow cytometry findings strongly suggest the need for novel approaches to maintain a healthy cell population in the presence of phenolics to achieve increased heterologous protein expression and, thereby, valorize lignin efficiently.

**Electronic supplementary material:**

The online version of this article (10.1186/s13068-018-1179-8) contains supplementary material, which is available to authorized users.

## Background

Lignin, an alkyl-aromatic polymer comprising 15–40% weight of the plant biomass, is generated in large quantities as a byproduct from the pulp and paper industry and also from the second-generation biofuel industry [1, 2]. Its rich aromatic carbon content makes it an attractive renewable resource for the production of valuable materials, chemicals, and alternatives to fossil fuels [3–5]. However, lignin has thus far been underutilized; the most common current application is combustion of the solid-phase residue for its thermal energy content [6]. Lignin valorization based on lignin-degrading microbes and enzymes can contribute to more efficient and environmentally benign use of lignin for sustainable production of value-added chemicals [4]. However, lignin is highly recalcitrant to microbial attack due to the presence of phenylpropanoid aryl-C_3_ units cross-linked via C–C and C–O bonds—its chemical heterogeneity further complicates the problem [7]. Metabolic pathway engineering is emerging as a successful route to valorize lignin for the production of valuable renewable chemicals such as vanillin and *cis, cis*-muconic acid, which can serve the food, flavor, plastic, and adhesive industries [8–10]. Synthetic biology tools such as promoters, ribosome-binding sites, terminators, and ribozymes for the regulation of biological modules are essential for the development of an efficient metabolic engineering chassis for these applications [11, 12].

**Fig. 1:**
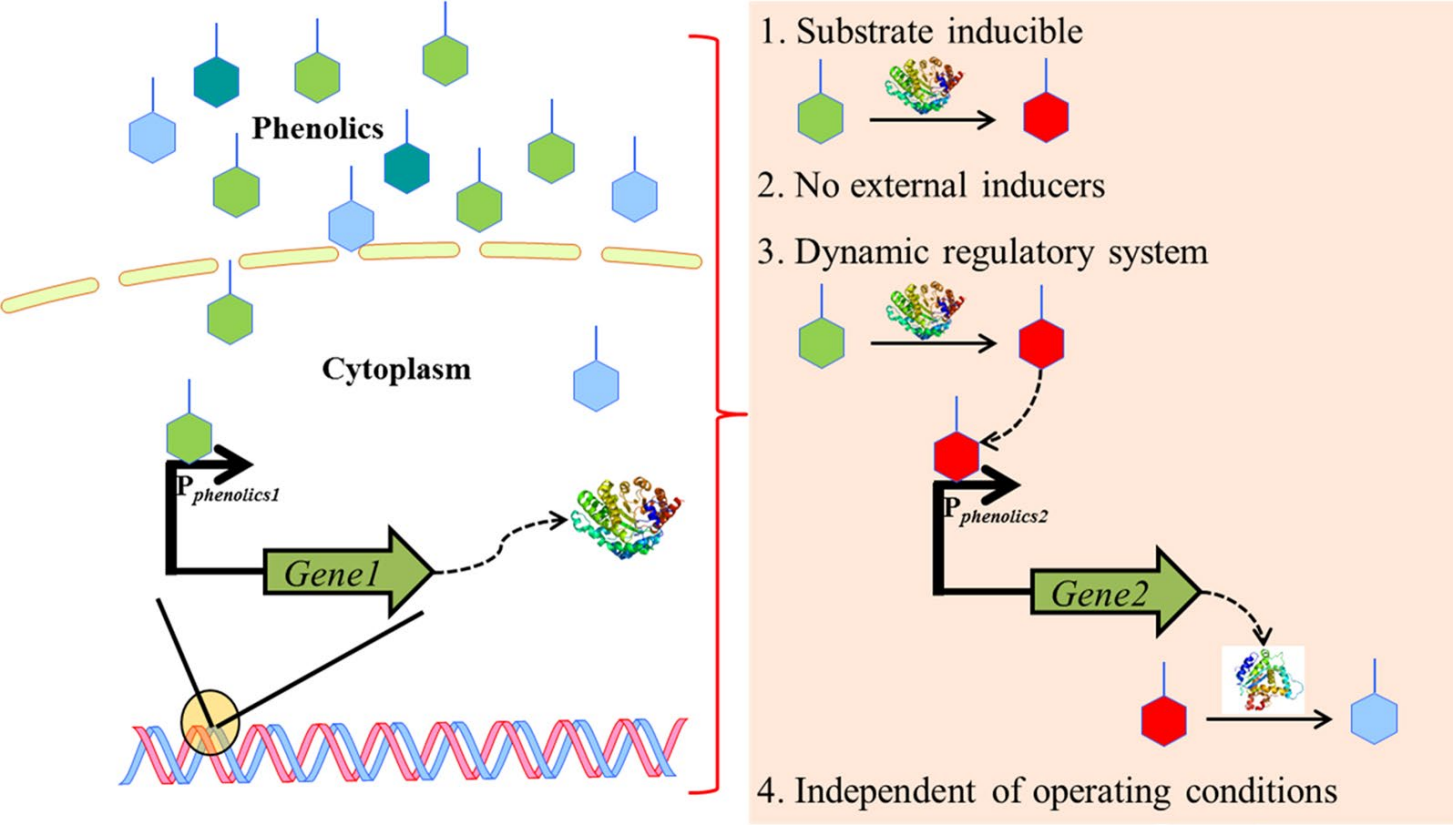
Lignin valorization chassis engineered with phenolic-inducible promoter. Promoters inducible by phenolics have the advantages listed on the right-hand side of the figure when employed for microbial lignin valorization

The most promising route to increase flux for product synthesis is to regulate or increase protein expression at the transcriptional level [12–14]. Promoter engineering enables discovery of additional synthetic promoter elements that lie beyond the endogenous promoters and allows tunable control of gene expression. Therefore, constructing novel synthetic promoters through promoter engineering is critical to aid lignin valorization efforts. Promoters inducible by phenolics provide the following advantages in a lignin valorization chassis (Fig. 1): (1) the engineered pathway can be designed to be activated in the presence of a lignin depolymerization product and catalyze the conversion of the same phenolic compound into its product. This leads to the development of substrate inducible systems; (2) external inducers such as IPTG or galactose are not required, reducing the overall process cost and the extra stress resulting from the external inducer [15]; (3) with the development of multiple phenolic-inducible promoters, a dynamic regulatory system, as depicted in Fig. 1, can be developed. A dynamic regulatory system avoids the buildup of toxic intermediates and saves carbon and energy for the cell which otherwise would be expended for protein synthesis, thereby, improving product yield, rate and titer [16, 17]; and (4) a phenolic-inducible promoter is independent of operation conditions, unlike other promoters that are inducible by environmental factors such as pH and temperature [18, 19]. Therefore, the system is more robust and provides flexibility in the selection of operation conditions.

In a prokaryotic promoter, the conserved hexameric − 35 element (TTGACA) and the − 10 element (TATAAT), also known as the Pribnow box, are the primary binding sites for the RNA polymerase [20, 21]. Improvement in promoter strength can be achieved by random mutagenesis of the entire promoter region [22, 23], saturation mutagenesis of nucleotide spacer regions [24–26], or through a hybrid promoter engineering approach [27–29]. Hybrid promoter engineering has resulted in the creation of powerful promoters that are being used by researchers around the world. *P*_tac_, which was developed in 1983, was the first of the widely used hybrid promoters [27]. In fact, *P*_tac_ along with its derivatives *P*_trc_ and *P*_tic_ are still commonly used today after more than 30 years of its discovery [30]. The use of these promoters for the expression of heterologous proteins is routine for *Escherichia coli*, *Bacillus subtilis* and *Synechocystis* 6803, among others [31–33]. Hybrid promoters based on the assembly of enhancer element and core promoter fusions have been successfully employed to improve the transcription efficiency or enable novel promoter regulation in eukaryotic systems as well [29, 34, 35]. This further demonstrates hybrid promoter engineering to be a promising and an efficient promoter engineering strategy for applications beyond prokaryotes. In this study, we have utilized a hybrid promoter engineering approach to create novel promoters that have improved gene expression in the presence of phenolics. The various aromatic compounds present in the lignin-rich liquor generated after the pretreatment of lignocellulosic biomass are listed in Table 1. Vanillin is one of the primary phenolic compounds explored as an inducer in this study, since it appears in majority of the streams generated from the lignin depolymerization (Table 1).

**Table 1:** Aromatic compounds generated from the depolymerization of lignin

Method	Depolymerization agent	Lignin source	Major products	Refs
Catalytic	Cu-CrO	Hardwood lignin	Methanol, 4-*n*-propylcyclohexanol, 4-*n*-propylcyclohexanediol, and glycol	[49]
	Mo	Kraft pine lignin	Phenol, cyclohexane, benzene, naphthalene, and phenanthrene	[50]
	H_3_PMo_12_O_40_	Kraft lignin	Vanillin and methyl vanillate	[51]
	CuSO_4_/FeCl_3_	Yellow poplar wood chips	Vanillin, syringaldehyde, acetovanillone, and acetosyringone	[52]
	CuO	Hardwood kraft lignin	Syringaldehyde, vanillin, syringic acid, and vanillic acid	[53]
Ionic liquid	[C2mim][OAc] at 160 °C	Kraft lignin, eucalyptus, switchgrass, pine	Guaiacol, vanillin, syringol, eugenol, and catechol	[5]
Microbial	*Aneurinibacillus aneurinilyticus*	Kraft lignin	Guaiacol, acetoguiacone, gallic acid, and ferulic acid	[54]
	*Bacillus* sp.	Kraft lignin	Ferulic acid, 3,4,5 trimethoxy benzaldehyde, and t-cinnamic acid	[55]
	*Rhodococcus jostii* RHA1 mutant	Wheat straw lignocellulose	Vanillin, 4-hydroxybenzaldehyde, and ferulic acid	[8]
	*Novosphingobium* sp. B-7	Kraft lignin	Ethanediol, *p*-hydroxybenzoic acid, and vanillic acid	[56]
Enzymatic	A β-*O*-4 linkage cleaving enzyme system (LigDFG)	Softwood alkali-lignin and hardwood alkali-lignin	Guaiacol, ferulic acid, eugenol, vanillin, and acetovanillone	[57]

Hybrid promoter engineering in general involves the fusion of two promoters comprising different characteristics, resulting in either a promoter of higher strength or an optimal promoter tailored to perform a specific function. The first step in this work was to identify an appropriate endogenous promoter that exhibits gene regulation in the presence of phenolics. The basal promoter from which the hybrid promoters for this study were developed was identified from an earlier research conducted by Strachan et al. [36]. Strachan and others interrogated the intergenic regions in *E. coli* leading to the discovery of the promoter PemrR which was found to be active in the presence of a few lignin derived monoaromatic compounds. In this study, towards diversifying the synthetic biology tools, three engineered promoters were constructed by swapping the spacer region of *P*_emrR_ to increase heterologous protein production in the presence of phenolics.

## Results and discussion

### Construction of hybrid promoters inducible by phenolics

**Fig. 2:**
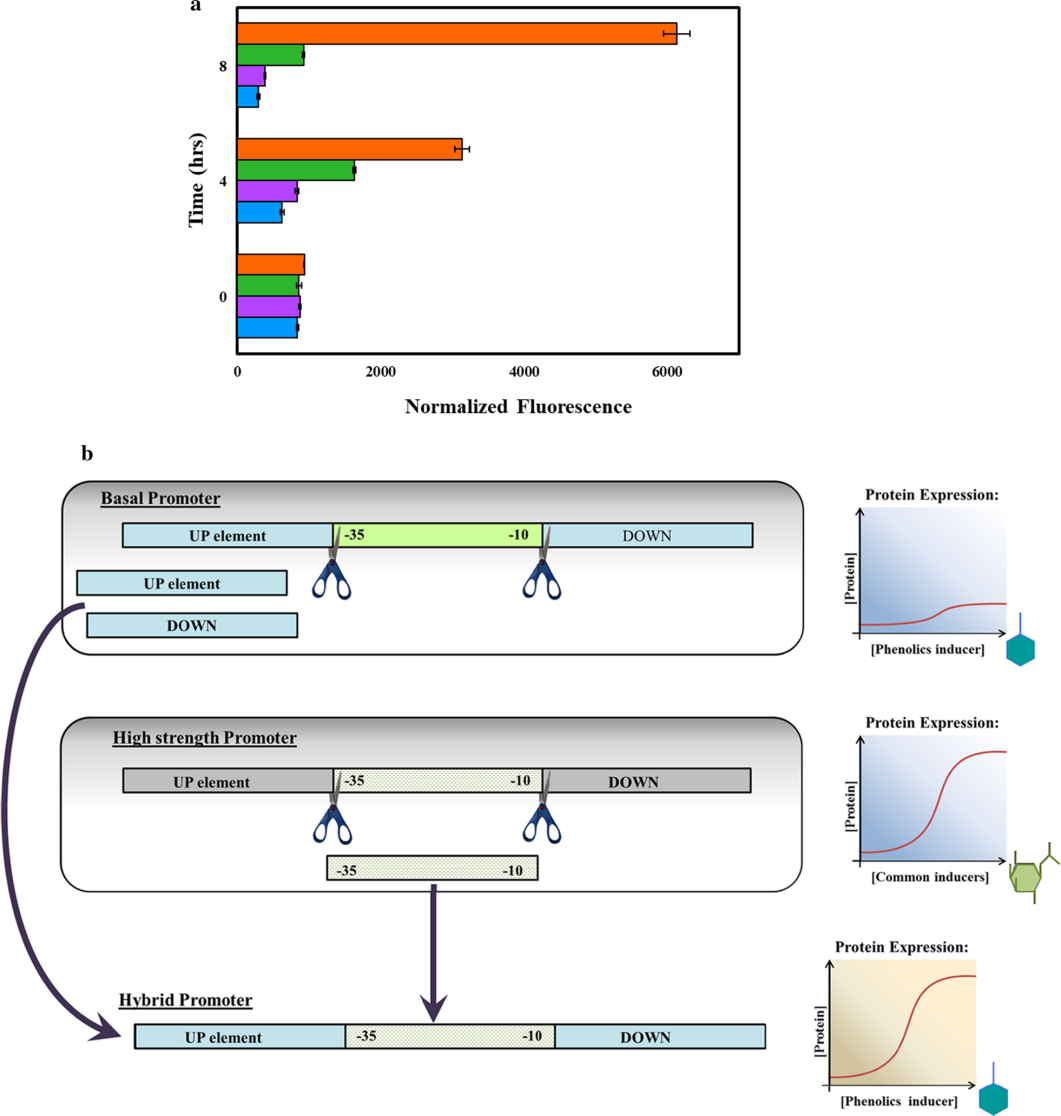
Construction of hybrid promoter to increase the strength of induction by phenolics. **a** Identification of a basal promoter inducible by phenolics. The promoter *P*_emrR_ is tested in the presence of vanillin by the expression of mCherry in *E. coli* and is found to be naturally inducible by vanillin that can be inferred by an increase in fluorescence at higher vanillin concentrations. 0 mM vanillin, blue bar; 0.1 mM vanillin, purple bar; 1 mM vanillin, green bar; 5 mM vanillin, orange bar. Each data represent the average of three biological replicates and the error bars represent standard deviation (s.d.); **b** Hybrid promoter engineering strategy for the construction of higher strength promoters inducible by phenolics. Swapping the spacer region from any stronger promoter with a phenolic-inducible promoter to increase promoter strength but retain inducibility

A biosensor was constructed to interrogate the activity of the promoter *P*_emrR_ by introducing a gene coding mCherry downstream of it and expressed in the *E. coli* strain Mach1. In addition, to verify that the promoter was active under the experimental conditions for this study, the strain RIF01 (Table 2) expressing *mCherry* under the control of *P*_emrR_ was tested in the presence of vanillin. Vanillin was chosen as the first phenolic compound to be explored; since it appears as a common substrate in most of the lignin, depolymerization methods employed (Table 1). Figure 2a confirms the induction of the promoter *P*_emrR_ with the addition of vanillin. In the presence of 5 mM vanillin, the fluorescence of the RIF01 strain increased by over 20-fold in comparison with cultures without vanillin (Fig. 2a). Therefore, *P*_emrR_ was selected as the basal promoter to be engineered for the construction of phenolic-inducible promoters of higher strength.

**Table 2:** Plasmids and strains

Plasmids/strains	Description	Source or reference
Plasmids		
pNW33N	Backbone plasmid for all vectors constructed in this study	Bacillus Genetic Stock Center (BGSC)
pRIF01	Derived from pNW33N with *mCherry* and the promoter, *P*_emrR_	This study
pRIF02	Derived from pNW33N with *mCherry* and the promoter, *P*_vtac_	This study
pRIF03	Derived from pNW33N with *mCherry* and the promoter, *P*_vtrc_	This study
pRIF04	Derived from pNW33N with *mCherry* and the promoter, *P*_vtic_	This study
Strains		
*E. coli* Mach1	Host strain	Invitrogen
RIF00	*E. coli* with pNW33N	BGSC
RIF01	*E. coli P*_emrR_::*mCherry*::Cm^r^	This study
RIF02	*E. coli P*_vtac_::*mCherry*::Cm^r^	This study
RIF03	*E. coli P*_vtrc_::*mCherry*::Cm^r^	This study
RIF04	*E. coli P*_vtic_::*mCherry*::Cm^r^	This study

The strength of a promoter is primarily dependent on the similarity of the hexameric elements (− 35 element and the − 10 element) to the consensus sequence along with the length and sequence of the spacer region in between [37, 38]. The sequences upstream and downstream of the spacer region have been known to contain activator and repressor-binding sites to either enhance or repress transcription of a gene in some bacterial promoters [39]. One of the previous successful efforts for engineering *E. coli* promoters involved fusing the enhancer element from different promoters to the core promoter of *P*_lac_ resulting in transcription increase by 1.5–90-fold [38]. However, given the lack of knowledge on the architecture of the endogenous promoter, *P*_emrR_, it is prudent not to disturb these sites on the first trial as they may interact with the phenolics or a phenolic bound complex to modify transcription. The spacer provides flexibility for the binding of the sigma factor and mutagenesis of the spacer region has been successful in increasing transcription in several cases [24]. Therefore, in this study as a new strategy to both diversify and increase the promoter strength, engineered promoters were created by importing the spacer regions from strong *E. coli* promoters and by fusing them with *P*_emrR_ (Fig. 2b). The promoters, *P*_tac_, *P*_trc_, and *P*_tic,_ were chosen to evaluate the effect of fusing their spacer regions into *P*_emrR_. In addition, the three promoters differ from each other by one nucleotide and have varying levels of gene expression (i.e., the strength of *P*_tac_ > *P*_trc_ > *P*_tic_) [30]. This work can be used as a proof of concept for the construction of more diverse engineered promoters from the spacers of other *E. coli* promoters if the same order of gene expression can be observed among the three engineered promoters.

### Vanillin as an inducer

**Fig. 3:**
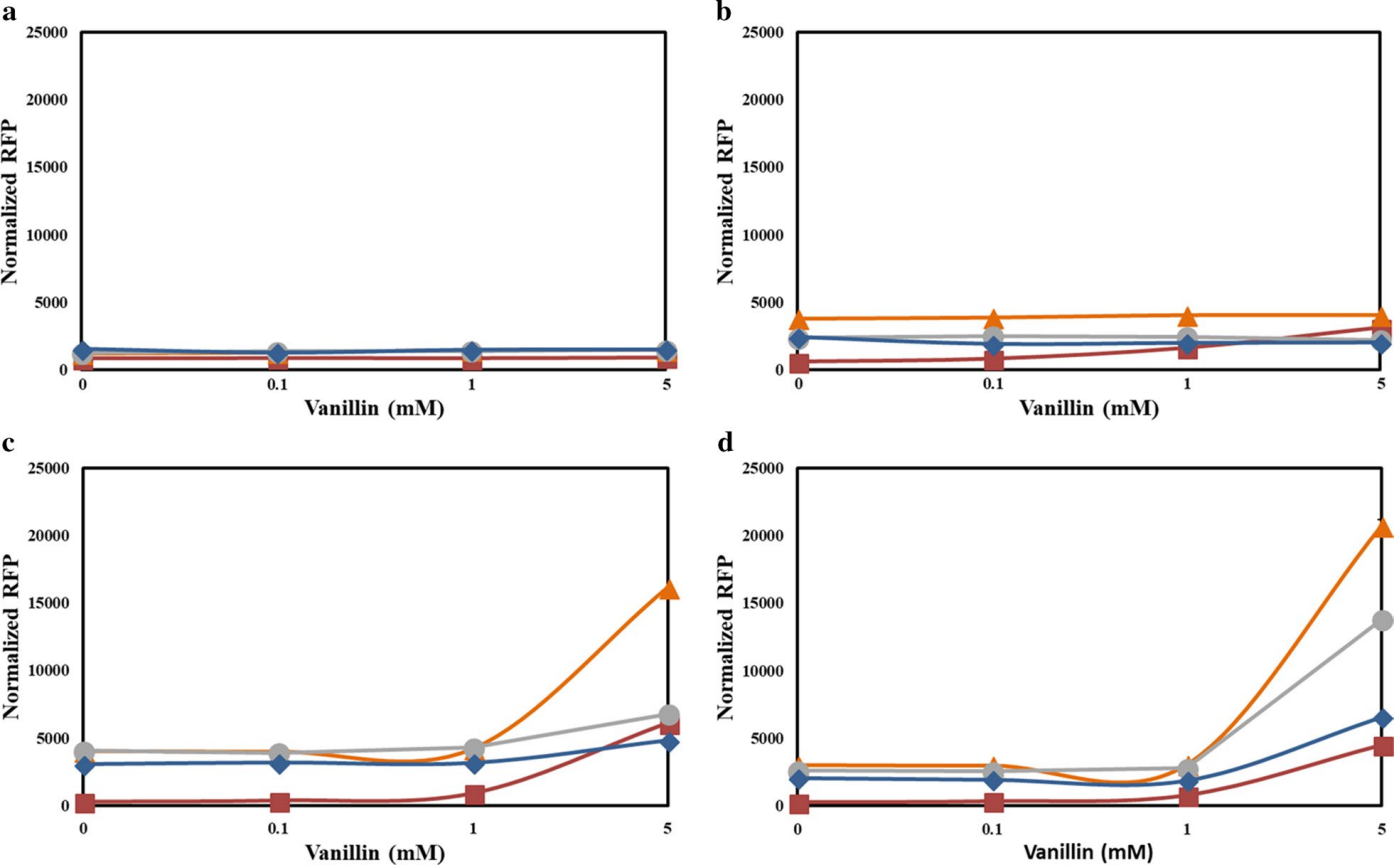
Performance of the engineered promoters in the presence of vanillin. Time after the addition of vanillin (inducer): **a** 0 h; **b** 4 h; **c** 8 h; and **d** 12 h. Gene expression is more prominent after 8 or 12 h after induction. The fluorescence of *E. coli* strains expressing *mCherry* under the native promoter *P*_emrR_ and the engineered promoters *P*_vtac_, *P*_vtrc_, and *P*_vtic_ were monitored using a fluorescence plate reader and normalized based on the cell density—OD_600_. The cells were grown in an M9 salt medium containing 25 mg l^−1^ chloramphenicol, 20 g l^−1^ glucose and 5 g l^−1^ yeast extract at 30 °C, and a 3 mm shaking amplitude. *P*_emrR_, red squares; *P*_vtac_, orange triangles; *P*_vtrc_, grey circles; *P*_vtic_, blue diamonds. Each data represent the average of three biological replicates and the error bars represent s.d. The standard deviations between the biological replicates were too small for the error bars to be visible

Engineered promoters *P*_vtac_, *P*_vtrc_, and *P*_vtic_ were constructed utilizing the strategy discussed in the previous section. To evaluate the performance of the engineered promoters, strains RIF02, RIF03, and RIF04 were constructed that had mCherry expressing downstream of the promoters *P*_vtac_, *P*_vtrc_, and *P*_vtic,_ respectively. The fluorescence measured from the promoters demonstrated that RIF02 carrying the engineered promoter *P*_vtac_ had the highest fluorescence 12 h after induction followed by the other strains with engineered promoters *P*_vtrc_ and *P*_vtic_ (Fig. 3). With respect to the control strain RIF01, the strains with the engineered promoters *P*_vtac_, *P*_vtrc_, and *P*_vtic_ had an increase in fluorescence by 4.6, 3.0, and 1.5-fold, respectively, when induced with 5 mM vanillin. This finding showed good correlation with the strength of the IPTG-inducible promoters from which the spacer sequences were obtained, i.e., strength of *P*_vtac_ > *P*_vtrc_ > *P*_vtic_. This offers the possibility that incorporation of the spacer region from other *E. coli* promoters can enhance the strength of the phenolic-inducible promoter. Fluorescence from the negative control strain, RIF00, containing an empty vector was 39-fold less in comparison with RIF01. This confirms that the background fluorescence from the culture due to the medium, vanillin, and *E. coli* is negligible. However, the experiment also revealed that the engineered promoters were highly leaky in comparison with *P*_emrR_. Even in the absence of vanillin, the normalized fluorescence of the strains RIF02, RIF03, and RIF04 was higher than RIF01. Therefore, further engineering studies are required on the flanking regions of the − 10 and − 35 motifs that may contain the activation and repression sites required to reduce the leaky expression of genes in the presence of engineered promoters.

### Promoter activity with other phenolics

**Fig. 4:**
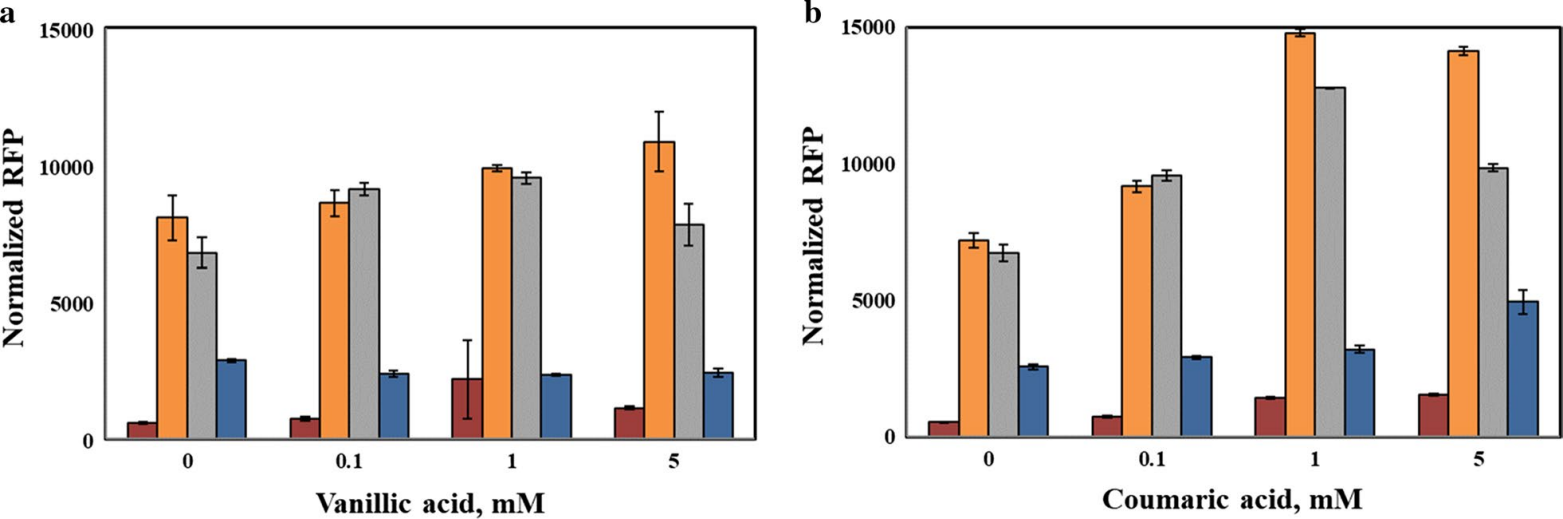
Performance of the engineered promoters in the presence of **a** vanillic acid and **b** coumaric acid. The fluorescence of *E. coli* strains expressing *mCherry* under the native promoter *P*_emrR_ and the engineered promoters *P*_vtac_, *P*_vtrc_, and *P*_vtic_ were monitored after 24 h of induction using a fluorescence plate reader and normalized based on the cell density—OD_600_. The cells were grown in an M9 salt medium containing 25 mg l^−1^ chloramphenicol, 20 g l^−1^ glucose and 5 g l^−1^ yeast extract at 30 °C and a 3 mm shaking amplitude. *P*_emrR_, red bars; *P*_vtac_, orange bars; *P*_vtrc_, grey bars; *P*_vtic_, blue bars. Each data represent the average of three biological replicates and the error bars represent s.d

The study was further extended to other phenolics including, vanillic acid, coumaric acid, guaiacol, and syringate by testing the strains RIF01, RIF02, RIF03, and RIF04 for their fluorescence emission. These phenolics were chosen, since they are closer in structure to vanillin, and at the same time, some of these compounds are also commonly present in the lignin depolymerized products, as listed in Table 1. However, not all of these phenolics were discovered to have gene regulation potential for the promoters that were tested. Vanillic acid and coumaric acid had some level of induction; nevertheless, it took a long duration (24 h) for the induction effect to emerge. The longer duration required for the induction could possibly be due to the transport limitation of the phenolics across the cell wall in comparison with vanillin. In comparison with *P*_emrR_, the engineered promoters *P*_vtac_, *P*_vtrc_, and *P*_vtic_ resulted in the improvement of expression levels by 9.5-, 6.8-, and 2.1-fold, respectively, with the addition of 5 mM vanillic acid (Fig. 4). Interestingly, the fluorescence at higher concentrations of vanillic acid and coumaric acid started to fall off slightly, especially for the strains engineered with the *P*_vtac_ and *P*_vtrc_ promoters despite no apparent drop in the OD600 of the cells at these concentrations. The OD600 of the cells at 24 h is shown in the Additional file 1: Figure S2. In the presence of 1 mM coumaric acid, the engineered promoters *P*_vtac_, and *P*_vtrc_, improved gene expression by 10.4- and 8.5-fold, respectively, whereas in the presence of 5 mM coumaric acid, the fold change for the promoters *P*_vtac_ and *P*_vtrc_ was 7.1 and 6.4, respectively, in comparison with *P*_emrR_. In addition, the basal level of expression for *mCherry* was higher for the engineered promoters (i.e., high leaky expression in the absence of the inducers vanillic acid and coumaric acid). This experiment also demonstrates that the promoter is highly specific towards vanillin and engineering the UP element of the promoter may result in altered specificity towards other phenolics.

### Flow cytometric analysis of the cell population

**Fig. 5:**
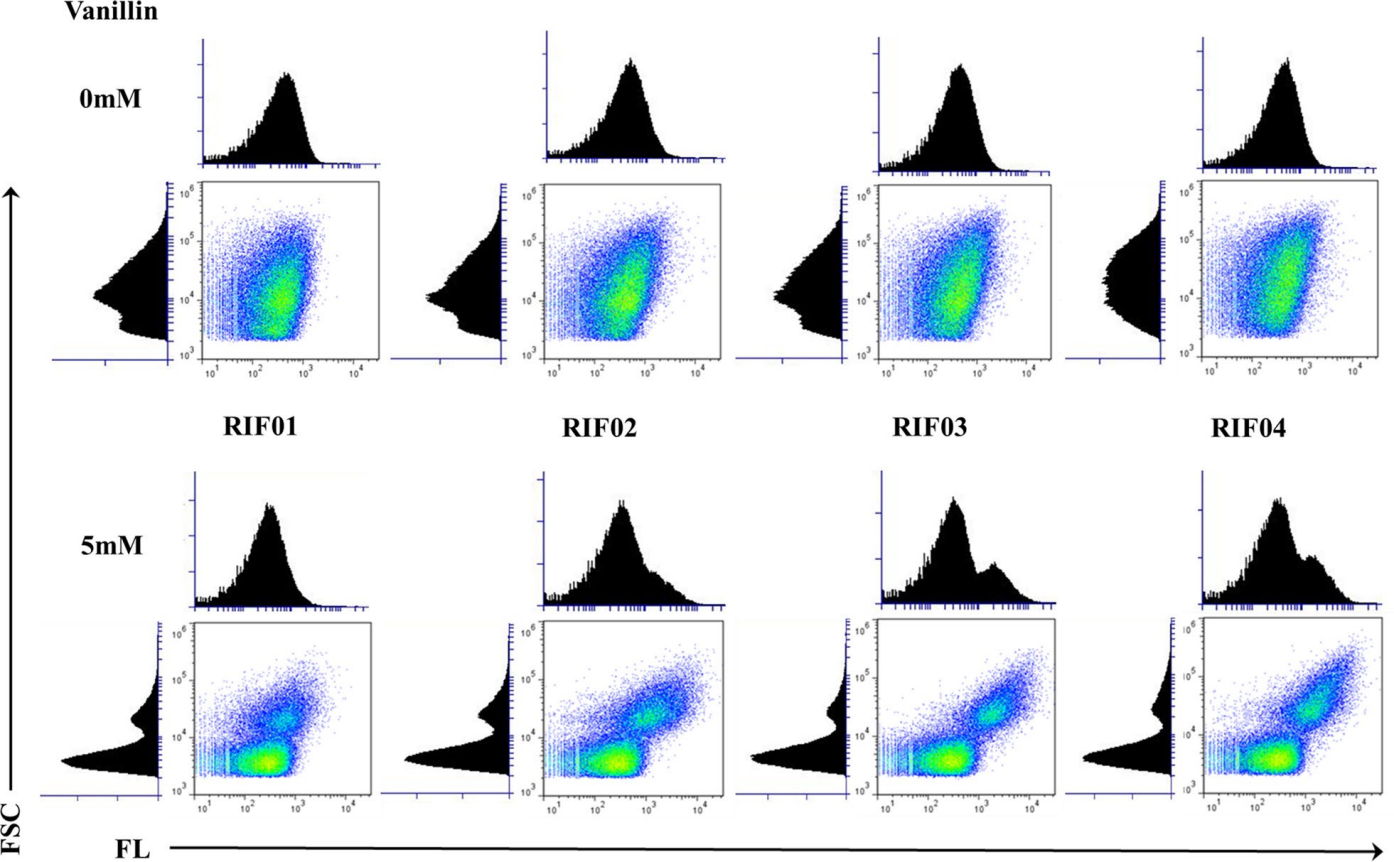
Flow cytometric analysis of vanillin-induced cultures. *E. coli* strains RIF01, RIF02, RIF03, and RIF04 contained promoters *P*_emrR_, *P*_vtac_, *P*_vtrc_, and *P*_vtic,_ respectively, for the expression of *mcherry*. Fluorescence (FL) was used as a measure of promoter strength, and forward scattering (FSC) was used as a measure of cell size

**Fig. 6:**
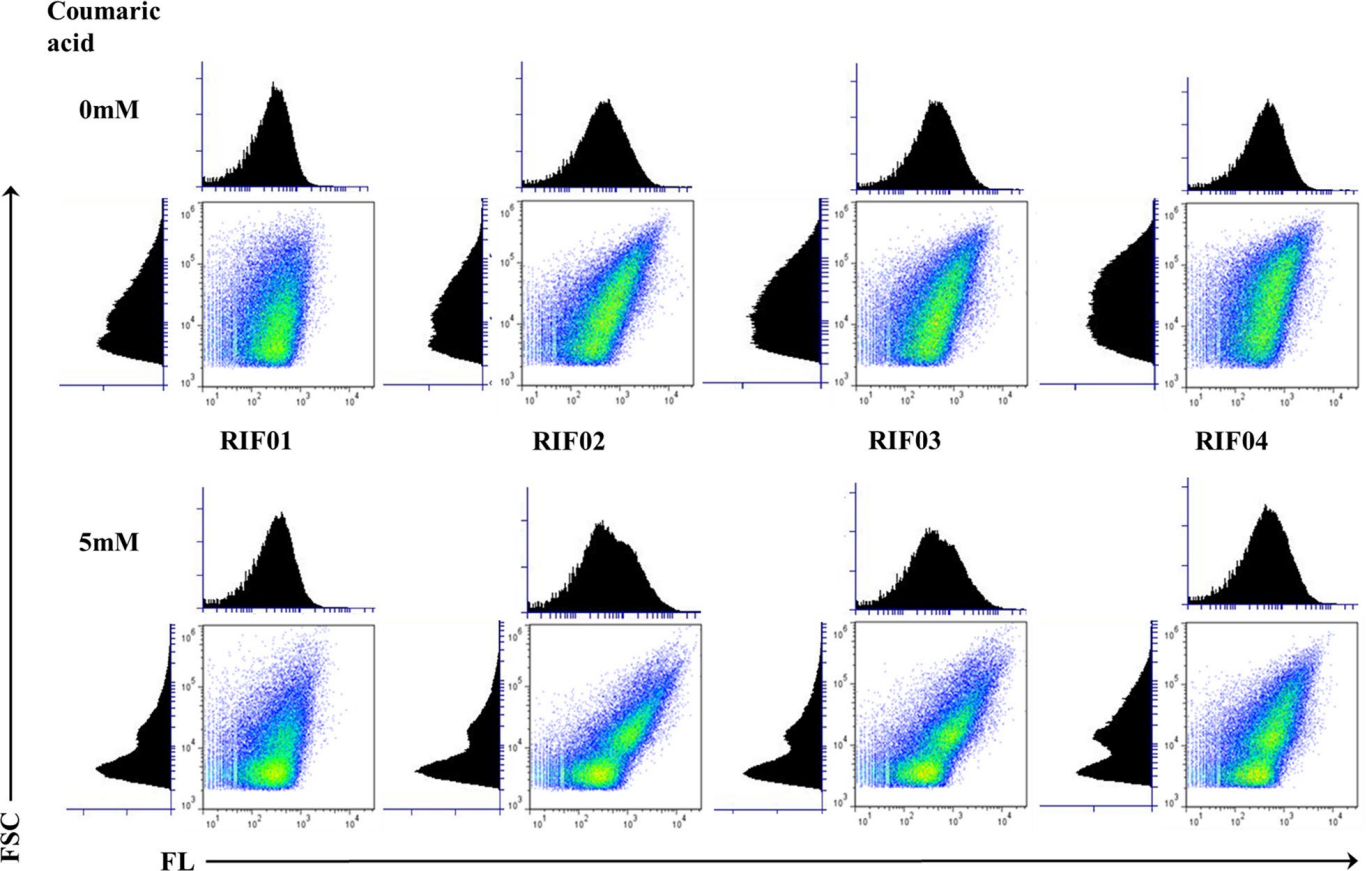
Flow cytometric analysis of coumaric acid-induced cultures. *E. coli* strains RIF01, RIF02, RIF03, and RIF04 contained promoters *P*_emrR_, *P*_vtac_, *P*_vtrc_, and *P*_vtic,_ respectively for the expression of *mcherry*. Fluorescence (FL) was used as a measure of promoter strength, and forward scattering (FSC) was used as a measure of cell size

The fluorescence results discussed thus far were obtained from a microplate reader and these values are not representative of the entire cell population, but an average behavior of all the cells. However, heterogeneity in gene expression can be present in the cell population due to several reasons. For example, a difference in gene expression may result among the cells due to either cell differentiation or morphogenesis [40]. Identifying population heterogeneity can help identify variables that negatively impact the gene expression and thereby aid in the development of robust expression systems. Flow cytometry is a technique that allows for the measurement of multiple physical and biological properties of single cells. Therefore, to analyze the heterogeneity within the cell population resulting from the induction of the promoters by phenolics, flow cytometry was employed in this study. Vanillin and coumaric acid-induced cultures were analyzed 24 h after induction for their fluorescence and light-scattering intensities with a flow cytometer. While intensity of fluorescence can be used as inference for protein levels, forward scattering can be used as a measure for the size of the bacterial cell. The correlation between forward scattering and fluorescence intensities among the different strains of *E. coli* with variation in vanillin and coumaric acid concentration is shown in Figs. 5 and 6, respectively. The flow cytometry figures for vanillin and coumaric acid concentrations in-between 0 and 5 mM are presented in Additional file 1: Figures S3, S4, respectively.

In agreement with our previous findings, the strains with the engineered promoters have a cell population that fluoresces much higher than the control strain, RIF01. In addition, a distinct sub-population of cells with higher forward scattering and higher fluorescence appeared for cultures that were induced with high concentrations of vanillin (greater than 1 mM vanillin). Light-scattering intensity has been considered to be roughly proportional to relative cell size and it has been observed that stationary phase cultures have a decreased cell size in comparison with exponential phase cultures [41]. A sub-population with lower forward scattering contained only background fluorescence, likely corresponding to the stationary phase cells with lower relative cell size. Interestingly, majority of the increase in fluorescence resulting from the engineered promoters was observed to come from the sub-population that had higher forward scattering. Furthermore, the heterogeneity within the cell population was not observed for *E. coli* cells that were induced with IPTG [42]. With coumaric acid, although highly fluorescent cells had higher forward scattering, a distinct sub-population did not appear as was the case with vanillin. To methodically identify the impact of promoter engineering on the heterogeneity of the cell population, the results were tabulated for cells with high forward scattering and high fluorescence (Additional file 1: Tables S3, S4). It can be verified from the table that in the presence of 5 mM vanillin, the highly fluorescent cell population increased from 5% in RIF01 to between 13 and 17% for the strains with engineered promoters (i.e., the strains RIF02, RIF03, and RIF04). However, the population of the cells with high forward scattering intensity remained about the same for all the promoters (i.e., between 24 and 27% of the total). These data suggest that there is no negative impact on the healthy and dividing cell population due to overexpression with the engineered promoters. In addition, the high forward scattering intensity population decreased from between 50 and 70% in the absence of vanillin to between 24 and 27% with 5 mM vanillin. These data along with the presence of sub-population in Fig. 5 suggest that vanillin stress is the likely cause for the observed heterogeneity. Therefore, the flow cytometry results suggest that the healthy population fraction needs to be maximized to achieve increased expression of the heterologous proteins.

### Promoter strength with variations in temperature

**Fig. 7:**
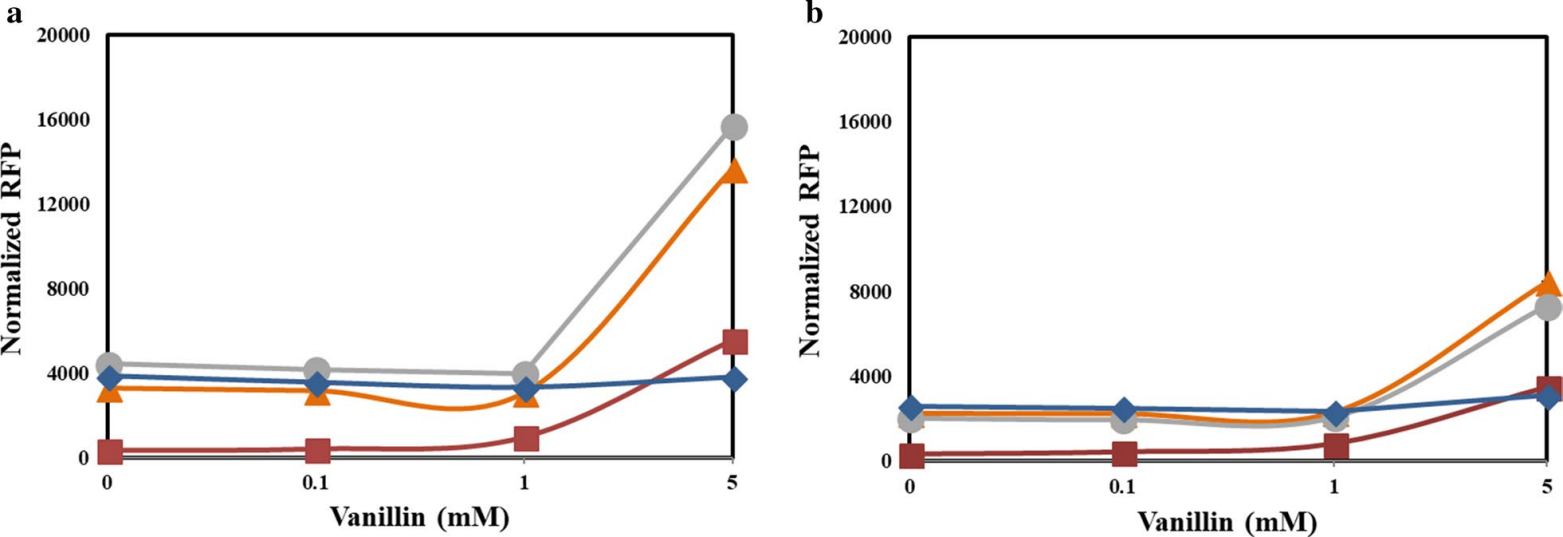
Performance of the engineered promoters at two different temperatures: **a** 37 °C and **b** 25 °C. The fluorescence of *E. coli* strains expressing *mCherry* under the native promoter *P*_emrR_ and the engineered promoters *P*_vtac_, *P*_vtrc_, and *P*_vtic_ were monitored after 8 and 24 h of induction at 37 and 25 °C, respectively, using a fluorescence plate reader and normalized based on the cell density—OD_600_. The cells were grown in an M9 salt medium containing 25 mg l^−1^ chloramphenicol, 20 g l^−1^ glucose and 5 g l^−1^ yeast extract at the corresponding temperatures and a 3 mm shaking amplitude. *P*_emrR_, red squares; *P*_vtac_, orange triangles; *P*_vtrc_, grey circles; *P*_vtic_, blue diamonds. Each data represent the average of three biological replicates and the error bars represent s.d. The standard deviations between the biological replicates were too small for the error bars to be visible

Temperature is a fundamental parameter in controlling the productivity of microbial fermentations, since each microbial species have their optimal growth temperature and the optimal temperature for the heterologous enzyme being expressed may vary. In addition, for certain heterologous expressions, low-temperature fermentations are preferred to ensure proper folding and thereby the function of the heterologous proteins [43, 44]. Therefore, to study the impact of operating temperatures on promoter strength, protein expression at two different operating temperatures, 25 and 37 °C, was examined. The cells were incubated at either of these temperatures after induction with vanillin instead of 30 °C that was regularly employed. From Fig. 7, it can be verified that the inducibility profile with vanillin has been retained at both these temperatures and this offers flexibility in operating temperatures for employing these promoters. At 37 °C, the mCherry expression profile obtained 8 h after induction (Fig. 7a) was comparable to the data obtained at 30 °C (Fig. 3). However, at 25 °C, the maximum normalized RFP values were reduced by half and were observed only 24 h after induction (Fig. 7b). This is likely due to the reduced growth rate of *E. coli* at lower temperatures. These experiments reveal that change in temperature does not impact the intensity of the expression strength for these promoters. Therefore, the microbial systems developed for lignin valorization with such engineered promoters can be operated at temperatures that are optimal for the microbe and the heterologous protein under study. We also envision that this work will serve as a springboard for the development of more promoters that will be inducible by a variety of other phenolics generated from lignin depolymerization and their subsequent deployment towards the development of an efficient lignin valorization chassis.

## Conclusions

In this research work, to demonstrate the strategy of swapping spacer region to modulate promoter strength while retaining inducibility, we have constructed three different phenolic-inducible promoters from a basal promoter. To our knowledge, this is the first research work that focuses exclusively on the development of promoters inducible by lignin-derived phenolics. By employing a hybrid promoter-engineering approach, three different engineered promoters were constructed by incorporating the spacer region of higher strength endogenous promoters in *E. coli*. In the subsequent experiments, we demonstrated that this engineering strategy resulted in significant improvements in the strength of the hybrid promoters. Therefore, this strategy should be generally applicable for improving the strength of engineered promoters by incorporating spacer regions from other high strength promoters. Furthermore, the strategy may also be employed to improve or diversify the strength of promoters that are inducible by other chemicals and factors. However, the engineered promoters were observed to be highly leaky, and therefore, reducing the leakiness of the promoters may require further engineering of the regions upstream and downstream of the − 10 and − 35 elements. The promoters were also observed to have a very long response time in the presence of vanillic acid and coumaric acid. We hypothesize that this could possibly be due to limitation in the transport of phenolics across the cell membrane. Therefore, studies on transporters and engineering host cells with transporters are critical for the development of an efficient microbial lignin valorization system.

Flow cytometry was employed to identify any heterogeneity in the cell population after induction with phenolics. The emergence of a sub-population constituting the metabolically active and dividing cells was observed especially in the cultures that were induced with 5 mM vanillin. In addition, this sub-population was identified as the major contributor for the heterologous protein that was expressed by the addition of phenolics as inducers. Therefore, further research effort will be required to increase the fitness of the strains in the presence of the phenolics which are known growth inhibitors at moderate-to-high concentrations. This aim can be achieved by applying the following techniques: (1) utilizing a rich media; (2) evolving the strains to improve their growth in the presence of the phenolics; and (3) engineering the strains with stress tolerance genes. This study should stimulate expansion of promoter engineering efforts to utilize cheap chemicals present in lignocellulosic biomass hydrolysates as inducers, potentially eliminating the need for common supplemental inducers such as IPTG, arabinose, etc.

## Methods

### Materials

Restriction enzymes, T4 DNA ligase, and plasmid miniprep kit were purchased from Thermo Fisher Scientific (Waltham, MA). Gel purification kit and Q5 polymerase were purchased from Promega (Madison, WI) and New England Biolabs (Ipswich, MA), respectively. All the reagents and cell culture media were purchased from Sigma-Aldrich (St. Louis, MO). Oligonucleotides were synthesized by Integrated DNA Technologies (Coralville, IA). The FluoSphere beads used for calibration of the flow cytometer were purchased from Invitrogen (Carlsbad, CA). The *E. coli* strain carrying the plasmid pNW33N was purchased from the Bacillus Genetic Stock Center (Columbus, OH). *E. coli* Mach1 purchased from Invitrogen was used for all the cloning and fluorescence experiments.

### Construction of plasmids and strains

The vector pNW33N containing a gene encoding for chloramphenicol resistance served as the backbone for all the plasmids constructed in this study. *mCherry* was PCR amplified from the plasmid pCtl-RFP-S_AraC_ [45]. The promoters to be tested were incorporated in the forward primers (Additional file 1: Table S1) that were employed for amplifying *mCherry*. Therefore, the DNA fragments obtained from this PCR amplification step had the promoters incorporated in the region upstream to the transcription initiation site of *mCherry*. The PCR obtained fragments were digested using the restriction enzymes *Bam*HI and *Hin*dIII. The digested fragments were purified and ligated into the same restriction sites of pNW33N using T4 DNA ligase. The ligation products were transformed into *E. coli* Mach1 cells using electroporation. The plasmids and strains constructed in this study to interrogate the strength of the promoters are listed in Table 1. Sequencing of the plasmid constructs was performed by Quintara Biosciences. A vector map of the plasmid constructed for interrogating the strength of the different engineered promoters is shown in Additional file 1: Figure S1.

### Cell growth and bulk fluorescence measurements

For the fluorescence experiments, frozen stocks of the strains were used to inoculate 5 ml of LB medium containing 25 mg l^−1^ chloramphenicol and incubated at 37 °C with an orbital shaking of 250 r.p.m. Overnight cultures were used to inoculate (0.1% volume/volume) an M9 salt medium containing 25 mg l^−1^ chloramphenicol, 20 g l^−1^ glucose, and 5 g l^−1^ yeast extract. The M9 salt medium (Sigma-Aldrich) contains 6.78 g l^−1^ Na_2_HPO_4_, 3 g l^−1^ KH_2_PO_4_, 0.5 g l^−1^ NaCl, 1 g l^−1^ NH_4_Cl, 0.1 mM CaCl_2_, and 2 mM MgSO_4_. 200 µl cultures of each strain (in triplicates) were loaded into black 96-well plates (black polystyrene plates with flat µclear bottom from Greiner Bio-One) and covered with Breathe-Easier sealing membrane (Sigma). The cultures were grown until mid-log phase at 37 °C and 250 rpm. The mid-log phase cells were induced with phenolics such as vanillin, coumaric acid, and vanillic acid at varying concentrations. The induced cells were incubated in a plate reader (Tecan Infinite 200 Pro) at 30 °C and a 3 mm shaking amplitude. The optical density of the cultures and the mCherry fluorescence was monitored at required intervals in the plate reader. Cell density was measured by monitoring the absorption of the cultures at 600 nm (OD600). Fluorescence was recorded using an excitation wavelength of 575 ± 9 nm and an emission wavelength of 610 ± 20 nm with a manual gain of 100 and a Z-position of 20,000 µm. Fluorescence readings are reported as normalized fluorescence given by the ratio of fluorescence of the cells to the OD600. The fold changes were reported as the ratio of fluorescence level of the strain with engineered promoter to the strain with the basal promoter.

### Flow cytometric analysis

Flow cytometric measurements were performed to study the heterogeneity amongst the *E. coli* cell population. The cells were transferred to a BD Accuri C6 Flow cytometer (Accuri Cytometers, Ann Arbor, MI) 24 h after induction with either vanillin or coumaric acid. The fluorescence emission from the cells was detected from all detector positions, and to study the expression of mCherry (Em-max = 610 nm), the signal from standard FL3 long-pass filter was utilized [46]. In addition, data were collected for the cells from forward scatter (FSC) and side scatter (SSC) channels. For all measurements, a flow rate of 14 µl min^−1^ was employed, corresponding to a core size of 10 µm. Calibrations were performed for scatter and fluorescence intensity using a set of fluorescently doped polystyrene beads with varying diameter (2.0, 7.52, 9.7, and 15.41 µm diameter) suspended in filter sterilized (0.22 µm, Millipore) de-ionized water [47, 48]. The primary threshold for an event was adjusted to a signal intensity value of 10,000 on FSC-H. Measurements were collected in triplicates from approximately 70 × 10^3^ cells per well to ensure statistical significance. The data generated by the flow cytometer were plotted and analyzed with FlowJo V7.

## Additional file

**Additional file 1: Table S1.** Oligonucleotides used in this study. **Table S2.** Nucleotide sequence of promoters used in this study. **Table S3.** Vanillin induced sub-population of cells with high fluorescence and forward scattering. **Table S4.** Coumaric acid induced sub-population of cells with high fluorescence and forward scattering. **Figure S1.** Vector map of the construct utilized to interrogate the strength of the promoters in this study. Based upon the promoter present in the construct, pRIFXX can be pRIF01, pRIF02, pRIF03, or pRIF04. **Figure S2.** Optical density of the *E. coli* strains under varying concentrations of vanillic acid and coumaric acid. **Figure S3.** Flow cytometric analysis of vanillin induced cultures. **Figure S4.** Flow cytometric analysis of coumaric acid induced cultures.
