# Supplementary material for: Hybrid phenolic-inducible promoters towards construction of self-inducible systems for microbial lignin valorization
Source: Biotechnol Biofuels. 2018 Jun 28;11:182. doi: 10.1186/s13068-018-1179-8 (PMC6022352; doi:10.1186/s13068-018-1179-8)
Supplement: Supplementary file 1 — Additional file 1: Table S1. Oligonucleotides used in this study. Table S2. Nucleotide sequence of promoters used in this study. Table S3. Vanillin induced sub-population of cells with high fluorescence and forward scattering. Table S4. Coumaric acid induced sub-population of cells with high fluorescence and forward scattering. Figure S1. Vector map of the construct utilized to interrogate the strength of the promoters in this study. Based upon the promoter present in the construct, pRIFXX can be pRIF01, pRIF02, pRIF03, or pRIF04. Figure S2. Optical density of the E. coli strains under varying concentrations of vanillic acid and coumaric acid. Figure S3. Flow cytometric analysis of vanillin induced cultures. Figure S4. Flow cytometric analysis of coumaric acid induced cultures. [file 13068_2018_1179_MOESM1_ESM.docx]

**Hybrid phenolic-inducible promoters towards construction of self-inducible systems for microbial lignin valorization**

**Table S1: Oligonucleotides used in this study**

| **Primer name** | **Sequence (5’→ 3’)** |
| --- | --- |
| Pr_f10a | gcgcggatccgaatcatttttctaaaacaatacatttactttatttgtcactgtcgttactatatcggctgaaattaatgaggtcatacccaaataaggaggatattatggtttccaagggcgaggag |
| Pr_f11a | gcgcggatccgaatcatttttctaaaacaatacatttgacaattaatcatcggctcgtataatgatcggctgaaattaatgaggtcatacccaaataaggaggggaattcatggtttccaagggcgaggag |
| Pr_f12a | gcgcggatccgaatcatttttctaaaacaatacatttgacaattaatcatccggctcgtataatgatcggctgaaattaatgaggtcatacccaaataaggaggggaattcatggtttccaagggcgaggag |
| Pr_f13a | gcgcggatccgaatcatttttctaaaacaatacatttgacaattaatcatcgcggctcgtataatgatcggctgaaattaatgaggtcatacccaaataaggaggggaattcatggtttccaagggcgaggag |
| Pr_f10b | cggctgaaattaatgaggtcatacccaa |
| Pr_r1c | gcgcaagcttgcggccgcagagtttgtagaaacgcaaaaaggcc |
| Pr_r1d | aagcttgcggccgcagagttt |
| Pr_f9 | gtcgatcggttcatcattcaccaaa |
| Pr_r2 | tgtgagttagctcactcattaggca |

**Table S2: Nucleotide sequence of promoters used in this study**

| **Promoter name** | **Sequence (5’→ 3’)** |
| --- | --- |
| **P_emrR_** | gaatcatttttctaaaacaatacatttactttatttgtcactgtcgttactatatcggctgaaattaatgaggtcatacccaaat |
| **P_vtac_** | gaatcatttttctaaaacaatacatttgacaattaatcatcggctcgtataatgatcggctgaaattaatgaggtcatacccaaat |
| **P_vtrc_** | gaatcatttttctaaaacaatacatttgacaattaatcatccggctcgtataatgatcggctgaaattaatgaggtcatacccaaat |
| **P_vtic_** | gaatcatttttctaaaacaatacatttgacaattaatcatcgcggctcgtataatgatcggctgaaattaatgaggtcatacccaaat |

**Table S3: Vanillin induced sub-population of cells with high fluorescence and forward scattering.**

| **Strain** | **Concentration, mM** | **Cell population %**  **(FL3-A >10^3^)** | **Cell population %**  **(FSC-A >10^4^)** |
| --- | --- | --- | --- |
| RIF01 | 0 | 2.91 ± 0.51 | 54.38 ± 4.47 |
|  | 0.1 | 2.87 ± 0.44 | 54.46 ± 1.65 |
|  | 1.0 | 2.09 ± 0.28 | 40.10 ± 2.17 |
|  | 3.0 | 2.22 ± 0.22 | 28.57 ± 0.48 |
|  | 5.0 | 5.18 ± 0.73 | 24.26 ± 2.98 |
| RIF02 | 0 | 8.62 ± 0.05 | 59.51 ± 1.67 |
|  | 0.1 | 6.37 ± 0.31 | 54.19 ± 2.99 |
|  | 1.0 | 7.08 ± 0.28 | 44.72 ± 1.97 |
|  | 3.0 | 8.68 ± 0.67 | 30.35 ± 0.56 |
|  | 5.0 | 13.52 ± 1.02 | 26.17 ± 2.44 |
| RIF03 | 0 | 7.81 ± 0.46 | 61.97 ± 0.41 |
|  | 0.1 | 6.21 ± 0.93 | 55.98 ± 1.29 |
|  | 1.0 | 6.48 ± 0.74 | 39.91 ± 1.73 |
|  | 3.0 | 8.58 ± 0.77 | 27.39 ± 0.47 |
|  | 5.0 | 16.89 ± 0.60 | 21.34 ± 0.50 |
| RIF04 | 0 | 7.61 ± 0.48 | 69.11 ± 0.39 |
|  | 0.1 | 7.21 ± 0.52 | 66.87 ± 0.72 |
|  | 1.0 | 7.48 ± 0.59 | 57.25 ± 2.04 |
|  | 3.0 | 15.67 ± 1.06 | 39.13 ± 2.03 |
|  | 5.0 | 18.22 ± 1.11 | 25.24 ± 1.21 |

**Table S4: Coumaric acid induced sub-population of cells with high fluorescence and forward scattering.**

| **Strain** | **Concentration, mM** | **Cell population %**  **(FL3-A >10^3^)** | **Cell population %**  **(FSC-A >10^4^)** |
| --- | --- | --- | --- |
| RIF01 | 0 | 3.34 ± 0.15 | 53.49 ± 0.87 |
|  | 0.1 | 3.07 ± 0.01 | 44.25 ± 0.71 |
|  | 1.0 | 3.87 ± 0.26 | 36.46 ± 0.51 |
|  | 5.0 | 4.01 ± 0.44 | 35.23 ± 3.34 |
| RIF02 | 0 | 20.88 ± 1.12 | 55.27 ± 1.47 |
|  | 0.1 | 24.90 ± 0.58 | 46.40 ± 1.81 |
|  | 1.0 | 24.37 ± 0.99 | 37.67 ± 0.55 |
|  | 5.0 | 21.43 ± 1.05 | 34.60 ± 1.45 |
| RIF03 | 0 | 16.67 ± 1.88 | 57.80 ± 2.17 |
|  | 0.1 | 20.49 ± 0.2 | 48.20 ± 1.31 |
|  | 1.0 | 22.95 ± 0.71 | 39.70 ± 0.89 |
|  | 5.0 | 21.62 ± 1.28 | 37.90 ± 1.40 |
| RIF04 | 0 | 12.93 ± 0.92 | 65.47 ± 0.38 |
|  | 0.1 | 12.58 ± 0.86 | 61.27 ± 0.15 |
|  | 1.0 | 14.45 ± 0.97 | 55.90 ± 0.87 |
|  | 5.0 | 15.85 ± 2.35 | 47.37 ± 2.99 |

**
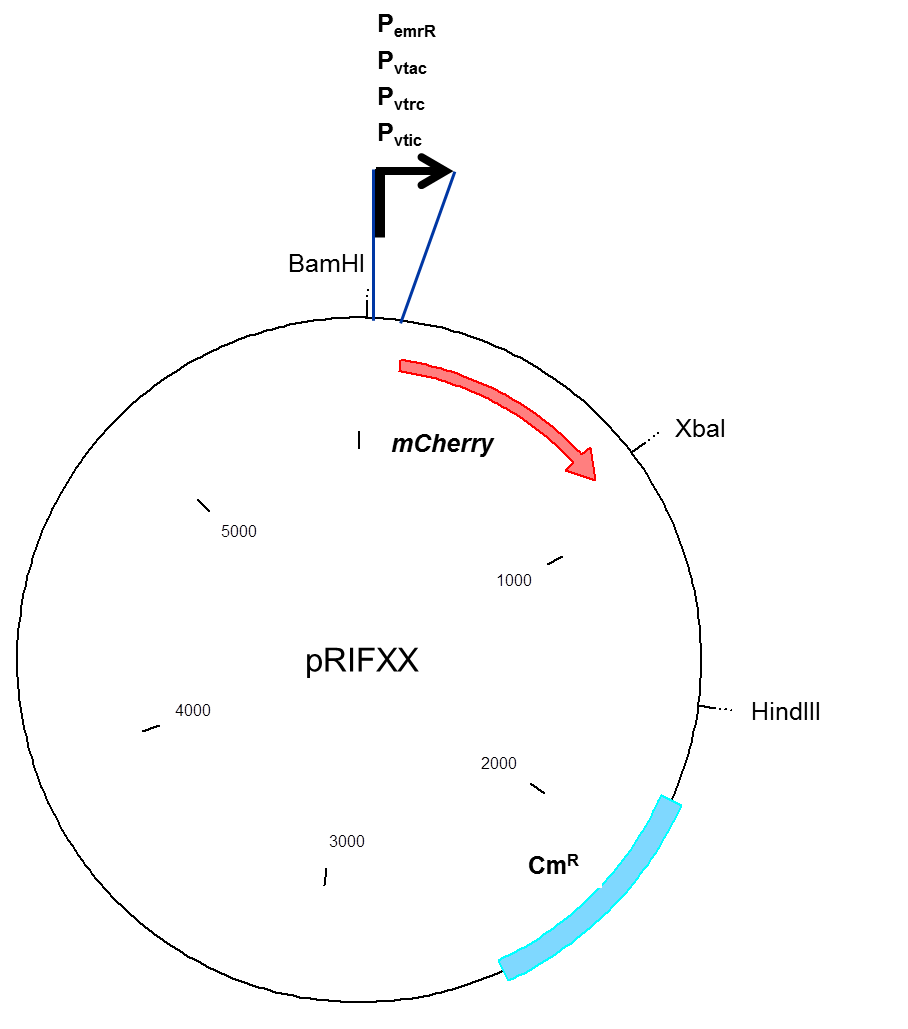
**

**Figure S1: Vector map of the construct utilized to interrogate the strength of the promoters in this study.** Based upon the promoter present in the construct, pRIFXX can be pRIF01, pRIF02, pRIF03, or pRIF04.

**
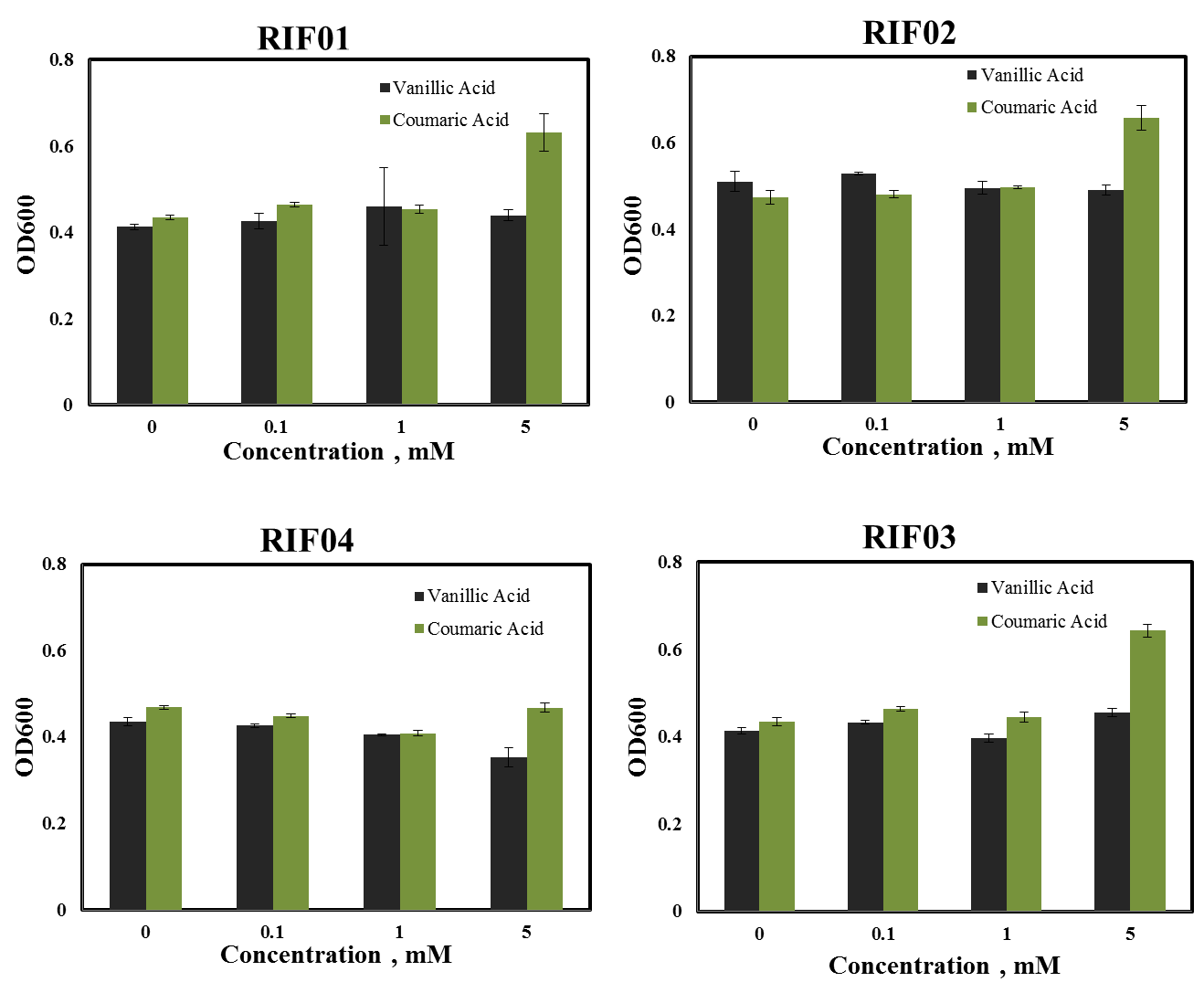
**

**Figure S2: Optical density of the *E. coli* strains under varying concentrations of vanillic acid and coumaric acid.**

**
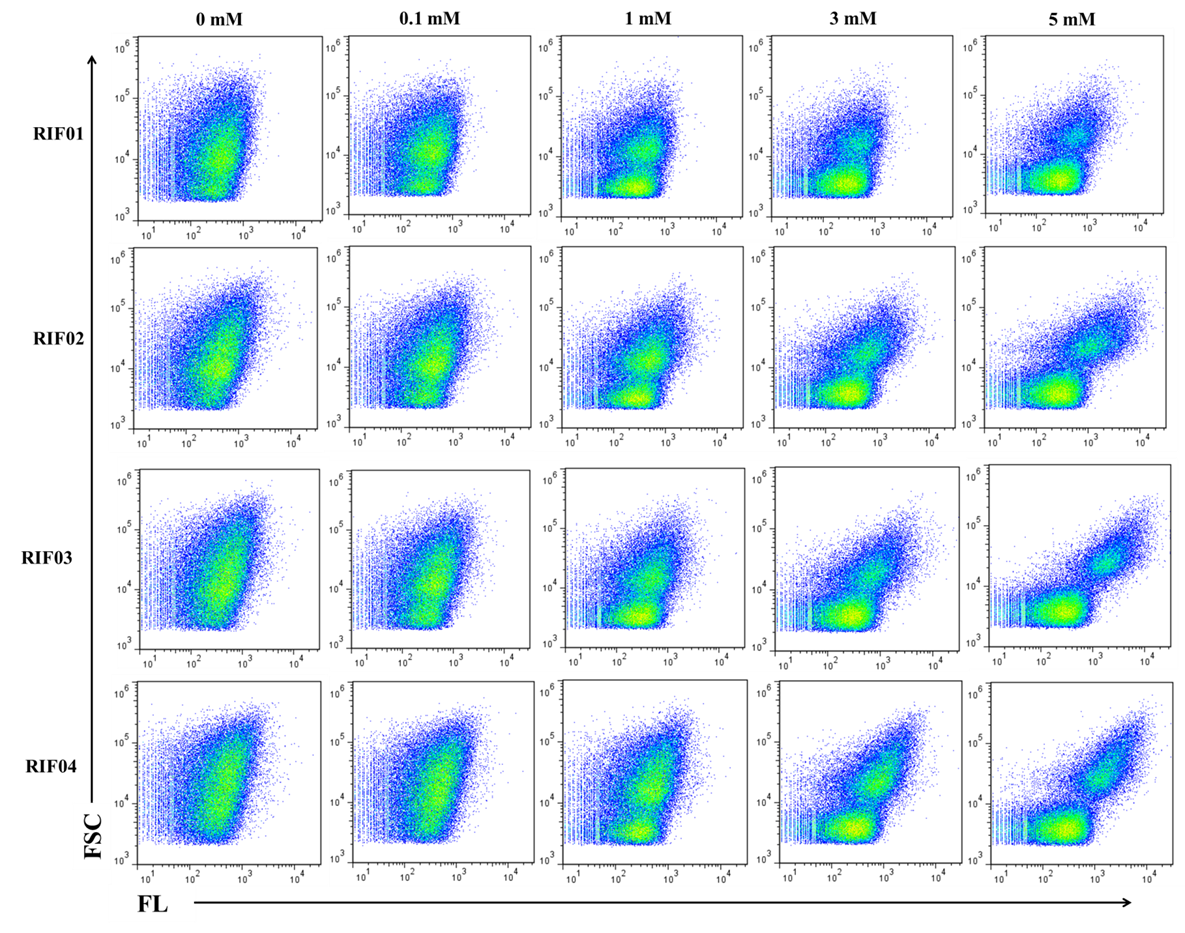
**

**Figure S3: Flow cytometric analysis of vanillin induced cultures.**

**
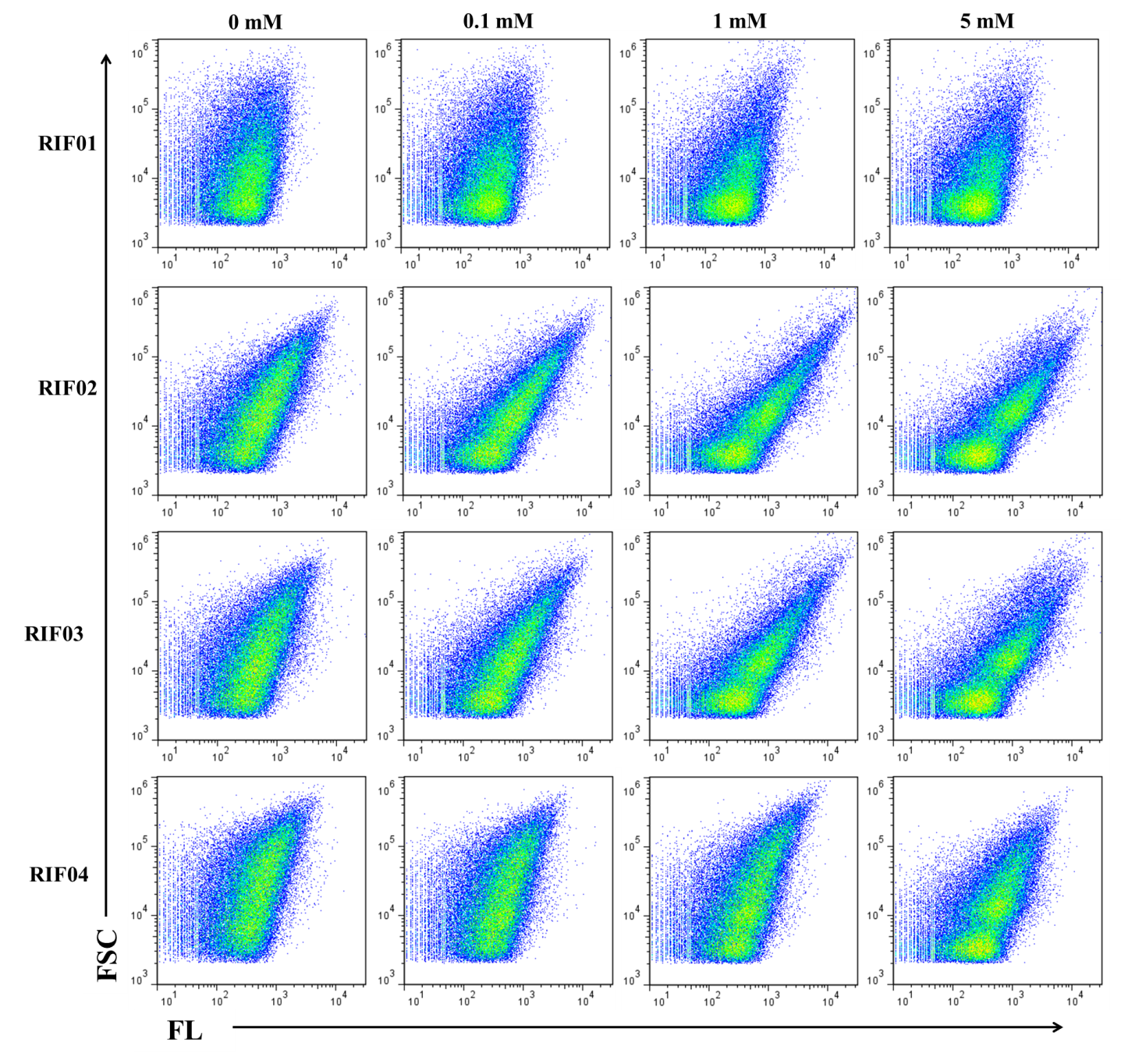
**

**Figure S4: Flow cytometric analysis of coumaric acid induced cultures.**
